# Food-Associated Calling in Gorillas (*Gorilla g*. *gorilla*) in the Wild

**DOI:** 10.1371/journal.pone.0144197

**Published:** 2016-02-24

**Authors:** Eva Maria Luef, Thomas Breuer, Simone Pika

**Affiliations:** 1 Max Planck Institute for Ornithology, Humboldt Research Group: Comparative Gestural Signalling, Eberhard-Gwinner-Strasse, 82319, Seewiesen, Germany; 2 Global Conservation Program, Wildlife Conservation Society, 2300 Southern Boulevard, Bronx, New York, 10460, United States of America; University of York, UNITED KINGDOM

## Abstract

Many nonhuman primates produce food-associated vocalizations upon encountering or ingesting particular food. Concerning the great apes, only food-associated vocalizations of chimpanzees (*Pan troglodytes*) and bonobos (*Pan paniscus*) have been studied in detail, providing evidence that these vocalizations can be produced flexibly in relation to a variety of factors, such as the quantity and quality of food and/or the type of audience. Only anecdotal evidence exists of eastern (*Gorilla beringei*) and western gorillas (*Gorilla gorilla*) producing food-associated vocalizations, termed singing or humming. To enable a better understanding of the context in which these calls are produced, we investigated and compared the vocal behavior of two free-ranging groups of western lowland gorillas (*Gorilla g*. *gorilla*) at Mondika, Republic of Congo. Our results show that (a) food-associated call production occurs only during feeding and not in other contexts; (b) calling is not uniformly distributed across age and sex classes; (c) calls are only produced during feeding on specific foods; and (d) normally just one individual gives calls during group feeding sessions, however, certain food types elicit simultaneous calling of two or more individuals. Our findings provide new insight into the vocal abilities of gorillas but also carry larger implications for questions concerning vocal variability among the great apes. Food-associated calls of nonhuman primates have been shown to be flexible in terms of when they are used and who they are directed at, making them interesting vocalizations from the viewpoint of language evolution. Food-associated vocalizations in great apes can offer new opportunities to investigate the phylogenetic development of vocal communication within the primate lineage and can possibly contribute novel insights into the origins of human language.

## Introduction

Many species of birds and mammals produce vocalizations upon encountering or feeding on certain foods (see, e.g. [1–3]). These so-called “food-associated vocalizations” are defined as vocalizations produced in the feeding context [4, 5]. The different degrees of informational content which are conveyed in the vocalizations range from simple advertisement of the presence of food in general [6] to specific information concerning certain characteristics of the food source [7–9] or characteristics of the specific ecological context of the food source [10]. In many nonhuman primate species (hereafter “primates”), it has been shown that food-associated vocalizations may convey information regarding the quantity [11, 12], quality [4], or divisibility of food [13]. Social factors may also influence calling behavior; for example, audience composition, especially in terms of the presence/ absence of close kin or closely affiliated individuals, influences decisions to call or not [14–16] and food-associated calls may function to regulate spatial patterning during feeding [7].

Concerning our closest living relatives, the great apes, only food-associated vocalizations of chimpanzees and bonobos have been systematically studied (see [17], for an overview). Recently, researchers showed that both chimpanzees and bonobos acoustically vary their calls to denote specific food items and that this information is meaningful to listeners [18, 19]. It has been argued that these ‘food-specific vocalizations’ are labels for specific food items and thus may represent evolutionary precursors to the phenomenon of linguistic reference in human language [17, 20].

While some researchers argue that acoustic differences in food-associated vocalizations merely reflect differences in emotional states during various food conditions [21, 22], there is increasing evidence that food-associated calls may be a class of primate vocalizations that display a greater degree of flexibility as compared to other vocalizations [16]. Evidence of vocal learning of food-associated calls in chimpanzees [23] supports this view of food-associated calls as more flexible vocalizations [10, 16]. Food-associated vocalizations are starting to attract increased research attention as they represent examples of a primate call class where vocal constriction may have relaxed, permitting certain evolutionary dynamics (such as learning) to act upon form and function. Therefore, food-associated vocalizations in great apes offer new possibilities to investigate the phylogenetic development of vocal communication within the primate lineage and can possibly contribute novel insights into the origins of human language.

Concerning gorillas, few reports exist describing their food-associated vocalizations. Schaller [24], Fossey [25] and Harcourt and colleagues [26] reported the occurrence of food-associated vocalizations in wild mountain gorillas and referred to them as singing and/or humming (also sometimes referred to as high hums), emphasizing the pure-tone acoustical elements that may resemble melodic bird songs and music. In addition, recent studies of the vocal repertoire of wild western lowland gorillas also noted singing and humming in the food context but did not provide more detailed descriptions of the behavior [27, 28]. humming in gorillas has also been described as a sign of contentment during feeding and possibly resting situations (however Fossey [1972] subsumed a number of close calls, including humming, together as *belch vocalizations* and it is unclear which of those is used during resting) or as a general signal of emotional well-being in a captive gorilla [29]. Detailed studies of the use of the vocalization, however, have not been conducted. Gorilla food-associated calls represent an interesting type of ape vocalization that is not only important in the context of the vocal repertoire of gorillas but also in the larger context of the vocal variability within the great apes.

The aim of the present study is to provide a preliminary understanding of gorillas’ food-associated calls by investigating their production with a special focus on the following four questions: (1) Is food-associated calling in gorillas strictly related to feeding or does it occur in other behavioral contexts as well? (2) Is food-associated calling uniformly distributed across age and sex classes? (3) Are food-associated calls in the feeding context produced in response to feeding on distinct foods? This includes firstly food types (leaves, fruit, stems/ pith, seeds, insects, flowers, and aquatic vegetation) and secondly preferred versus non-preferred (fallback) foods (sensu [30]). (4) Are concurrent calling bouts related to the food source? In sum, our study aims to determine the relationship of gorilla food-associated calls to context, social characteristics of the vocalizer (sex, age), and the food source in order to gain insights into functional aspects of the vocalizations.

## Methods

### Ethics statement

The Wildlife Conservation Society granted field permission (together with Ministère de la Recherche Scientifique of the Republic of Congo & Ministère de l´Economie Forestière et Développement Durable, Republic of Congo) and ethically approved the study. The Max Planck Institute for Ornithology provided ethical approval to the study. The research adhered to the legal requirements of the countries in which it was conducted (Republic of Congo and Germany), to guidelines of UFAW, and to the principles of “Ethical Treatment of Non-Human Primates”, as stated by the American Society of Primatologists. The study was approved by the Wildlife Conservation Society (Ref. Nr. 084/ DV/ WCS-12) and the Ministère de l´Economie Forestière et Développement Durable and authorized by the Ministère de la Recherche Scientifique (Délégation Générale de la Recherche Scientifique et Technologique, Ref. Nr. 009/ MRS/ DGRST/ DMAST)

### Study area and species

Data were collected between July and September 2012 from two free-ranging, habituated groups of western lowland gorillas at the Mondika Research Centre in the Republic of Congo. The study site is located in the Djéké Triangle, a protected area in a logging concession bordering the Dzanga-Ndoki National Park (Central African Republic) and the Nouabalé-Ndoki National Park (Republic of Congo) (2° 21’ 859” N, 016° 16’ 465” E). The forest habitat has been described as mixed-species tropical lowland forest, with monodominant *Gilbertiodendron dewevrei* (Caesalpiniaceae) forest, and swamp forest [31]. The climate in the study area consists of a long dry season of less than 100 mm monthly rainfall between December and March and a total annual rainfall of around 1600 mm with peaks between September and November [32]. From July to September fruit availability shows its annual peak in the Northern Congo and frugivory is significantly increased during that time whereas consumption of less preferred leaves and stems is decreased [32]. Two gorilla groups were followed daily (either one group all day, or one group in the morning and the other in the afternoon) from 7am until 4:30 pm by EML and a research assistant with the help of three to five Ba’aka trackers. The groups consisted of eleven and thirteen individuals and were observed for approximately 450 and 150 hours, respectively (see Table 1 for details on the groups, including age and sex characteristics).

**Table 1:** Compositions of the two habituated gorilla groups at Mondika. Silverbacks Kingo and Buka are unrelated. Group Kingo was observed for approximately 450 hours; Group Buka was observed for approximately 150 hours.

Individual and group	Age and sex class
Kingo, Group Kingo	Silverback male
Mekome, Group Kingo	Adult female
Mama, Group Kingo	Adult female
Emily, Group Kingo	Adult female
Ugly *, Group Kingo	Adult female
Makombe, Group Kingo	Nulliparous adult female
Ndoki Bai *, Group Kingo	Nulliparous adult female
Kusu, Group Kingo	Subadult male
Ekendi, Group Kingo	Juvenile male
Itephi, Group Kingo	Infant male
Camarra, Group Kingo	Infant male
Kenga, Group Kingo	Infant male
E. K., Group Kingo	Infant male
Buka, Group Buka	Silverback male
Mama Passa *, Group Buka	Adult female
Yamilie, Group Buka	Adult female
Mobimba, Group Buka	Adult: Young silverback male
Balema, Group Buka	Adult: Blackback male
Fang, Group Buka	Subadult male
Diarlu, Group Buka	Subadult female
Gremlin *, Group Buka	Subadult female
Koloka, Group Buka	Juvenile female
Matula, Group Buka	Juvenile female
Paki Paki, Group Buka	Juvenile male

Asterisks indicate that individuals were excluded from the study due to a lesser habituation degree or because of disappearance during the field season.

Group Kingo has been habituated to human observers since the late 1990’s (see [33]) and was observed at a distance of approximately 7 to 10 meters. The habituation of group Buka started in 2008 and the group was less well habituated which meant that recording distance was (with the exception of the silverback male) between 8 and 12 meters. Nonetheless, the majority of individuals in group Buka could be classified as fully habituated (i.e. stage 5, see [33]). While the silverback males and immature individuals in each group were very well habituated, some females remained at the periphery of their group and were thus largely unhabituated to human observers. Those individuals were excluded from the study (indicated with an asterisk in Table 1) as their behavior was not comparable to the other group members due to their different degree of habituation. We compared food-related vocalizations only of individuals that showed a similar degree of habituation to humans (stage 5 of the habituation process, see [33]). Age classes of gorillas were defined according to Breuer et al. [34].

### Data collection

**Fig 1:**
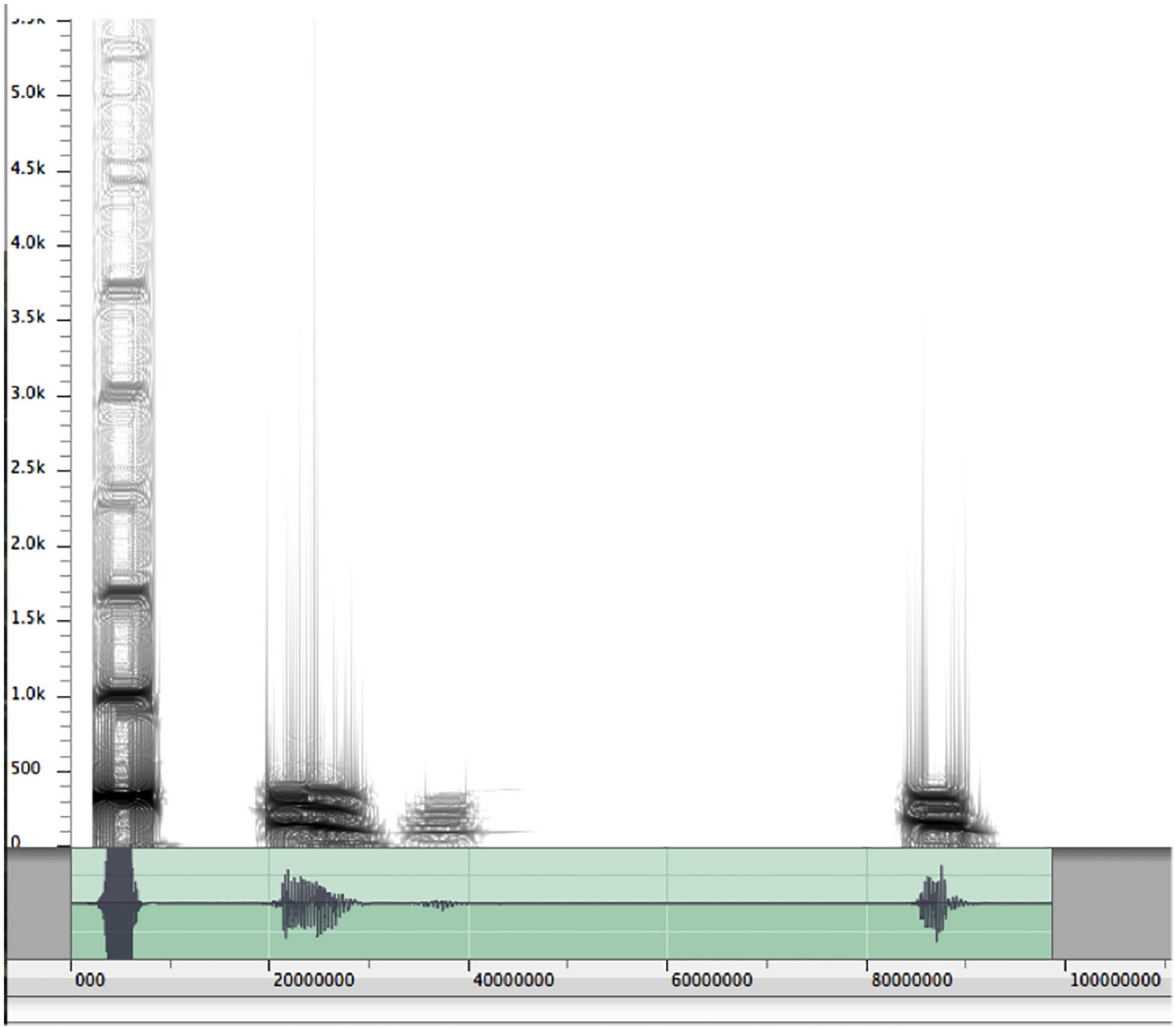
Spectrogram of singing.

**Fig 2:**
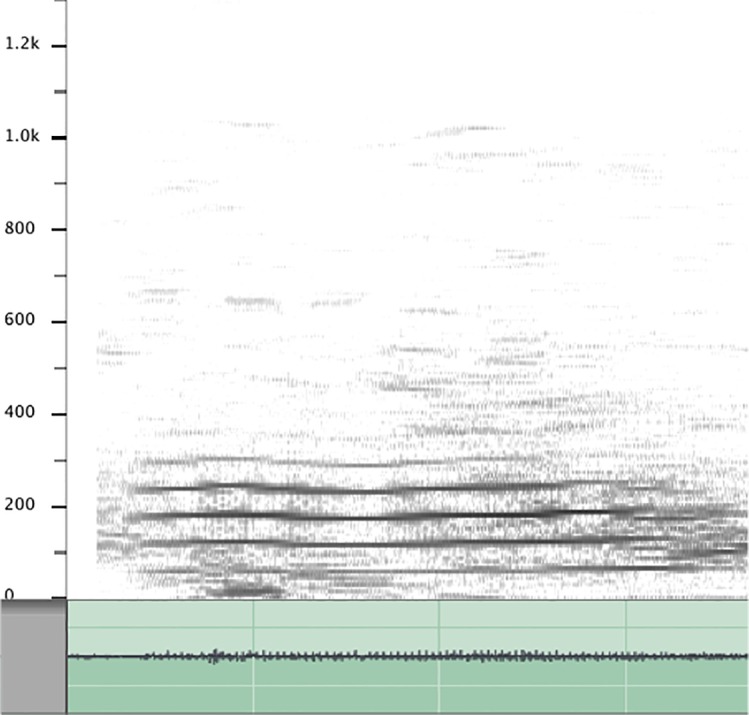
Spectrogram of humming.

Food-associated vocalizations of western lowland gorillas consist of two vocal types: (1) humming and (2) singing (see [27]). While humming is characterized by a prolonged and tonal low-frequency sound, singing is characterized by the utterance of multiple short and differently pitched sounds in close succession (also see [27, 28]) (see Figs 1 and 2 for spectrographic depictions of the two vocal types; audio samples of both vocalizations can be found in the supporting information, see S1–S4 Files). On occasion, there may be mixed food-associated calls, consisting of both singing and humming parts (Luef, personal observation).

The food-associated calls of gorillas are rather quiet and recordings are easily corrupted by forest noises (Luef & Breuer, personal observation). Individuals often brushed up against vegetation or moved on dry leaves while eating, causing loud interference noises, or they would turn their backs to the microphone. This precluded more detailed spectrographic analyses, as in most of our recordings the signal-to-noise ratio was not sufficient for in-depth acoustical investigations. The recorded food-associated calls were thus classified as singing or humming by ear. A second independent researcher coded 20% (N = 60 food-associated calls) of the data in order to ensure reliability of coding; the two raters agreed on the two coding variables with a kappa value of 0.84, which is considered “very good agreement” [35]. Specific acoustical distinctions of the humming and singing vocalizations can be found in Salmi et al. [27] and Hedwig et al. [28].

As humming and singing were rare events, we conducted behavior sampling and aimed to collect these behaviors on an all-occurrence basis from the whole group being followed. When the group fed in a single feeding patch, it was possible for observers to be confident that all of these soft vocalisations would be detected and thus the data was sampled on an all-occurrence basis [36]. In non-feeding contexts, it was not possible to reliably detect these soft vocalisations from the whole group, so sampling in these contexts was ad-libitum [36]. During feeding contexts, continuous audio recording of the group was taken. In addition, scan samples of vocal behaviour during feeding (food-associated calling yes/ no) were taken every 5 minutes.

For each recorded food-associated call, we noted the following parameters: (a) identity of vocalizer, (b) behavioral context, and if feeding context, we also recorded (c) the food species being eaten by the group and vocalizer (for food classification see [31]). The behavioral context was broadly defined by the activity of the majority of adult group members and the particular vocalizer (i.e. feeding, travelling, resting, socializing) [37]. One call was defined as continuous sound production delineated by pauses of more than 10 seconds. After a 10 second pause, a new call instance was recorded.

A feeding session was defined whenever all members of the group were feeding on the same food item. In that case, the entire group had to feed in one food patch and on one food species (to ensure the same ecological condition for each individual). A food patch was defined as an area of not more than approximately 15m^2^ in which all members of the gorilla group were present and feeding on the same food type. This excluded some of the overall feeding data of the gorillas as they often fed on dispersed foods, with the individuals widely dispersed and not all engaged in feeding or not all feeding on the same food. Since such dispersed instances did not allow us to define a clear feeding patch, we did not consider those feeding sessions. Therefore, patchy resources, such as leaves and fruit, represent the majority of our data. An additional fact is that our data collection period coincided with the main fruiting season at Mondika thus resulting in high rates of frugivory recorded. When feeding activity in one food patch had stopped and all group members started to engage in different activities (e.g. travelling, resting), the food bout was noted as finished. If feeding on the same patch was resumed at a later point, this was counted as a new food patch. Data on gorilla group feeding activities were collected with the help of a research assistant and an additional three to five field assistants (Ba’aka trackers) who helped scan the feeding patch to determine the relative spacing and activity of all group members.

Food species were identified with the help of the Ba’aka trackers and confirmed via photography by two botanist guidebooks for the region, one compiled by Angelique Todd (for the Bai Hokou Study Site, Central African Republic) and the other by Nasasha Shah (specifically for the Mondika study site).

Doran-Sheehy and colleagues [32] calculated preferred and fallback foods at Mondika according to a monthly availability and a monthly consumption score, reasoning that feeding on fallback foods would decrease when availability of preferred foods increased. Fallback foods of gorillas, including many leaves such as *Thomandersia hensii* and *Whitfieldia elongata*, were generally higher in lignin and were avoided when preferred foods higher in sugar and lower in fiber were available, such as the seeds of *Gilbertiodendron dewevrei* and the fruit of *Annonidium mannii* [32]. When available, we used the food preferences as suggested by Doran-Sheehy and colleagues to test whether the preference category of food has an impact on food-associated calling behavior.

To investigate the influences on food-associated calling in gorillas, we used Generalized Mixed Linear Models (GLMM, [38]) with logit link function and either binomial error structure (Models 1 and 3) or Poisson error structure (Model 2). Into this we included as the key test predictors the age classes (*adult*, *subadult*, *juvenile*, *infant*) and sex classes (*F*, *M*), the food types that were eaten during the feeding sessions (*fruit*, *leaf*, *insect*, *seed*, *stem*, *flower*, *aquatic vegetation*) and–for the cases for which available—the preference category of the specific foods (*fallback*, *preferred*) as well as the interaction as fixed effects. As random effects (intercepts) we included individual gorillas and feeding bouts. We used R (version 3.1.2, [39]) and lme4 [40] to perform linear mixed effects analysis models of the relationship between the fixed effects and (1) call per feeding bout (calling yes/ no), and (2) call rate (number of calls given per observed feeding hour per individual). In a third model, we investigated the probability of more than one individual calling per feeding bout (concurrent calls present/ absent) in relation to the fixed effects food types (*fruit*, *leaf*, *insect*, *seed*, *stem*, *flower*, *aquatic vegetation*) and preference category of the specific foods (*fallback*, *preferred*). Visual inspection of residual plots did not reveal any obvious deviations from homoscedasticity or normality. P-values were obtained by likelihood ratio tests of the full model with the effect in question against the model without the effect in question. The sample size for the models was 8603 observations, involving 826 food bouts, 327 food-associated calls and 20 individuals. The models testing food preference category in relation to the fixed effects displayed reduced sample sizes due to the facts that food preference category was not available for all food species. The sample size for the preference models was 4577 observations, involving 435 food bouts, 152 food-associated calls and 20 individuals.

## Results

### Contexts of food-associated calling

All instances of food-associated call production in gorillas were related to the feeding context; no ad-libitum data of humming and/ or singing were observed in any other context. We recorded a total of 327 food-associated calls (Group Buka: 152, Group Kingo: 175) during 827 feeding bouts (Group Buka: 257, Group Kingo: 580). 92% (N = 301) of the calls occurred during the actual feeding activity. The remaining 8% (N = 26) of food-associated calls were produced (a) after feeding had stopped and the vocalizer was moving away from the feeding patch either still chewing on or carrying the food item (N = 21), (b) while the vocalizer was sitting in a feeding patch but currently not eating while the other group members fed (N = 2), or (c) right before feeding started, i.e. when the vocalizer approached the food patch right before sitting down and feeding (N = 3).

In the majority of cases, one individual would call during a feeding bout (86%, N = 282). There were, however, instances when two individuals called simultaneously and produced partly overlapping food-associated calls (13%, N = 45), and rarely three individual calling at the same time (0.61%, N = 2). The concurrent calling bouts of two individuals consisted of (a) silverbacks and their male offspring (Group Buka: N = 6; Group Kingo: N = 31), (b) two siblings (Group Buka: N = 3), (c) silverback male and unrelated, adult female (Group Kingo: N = 4), and (d) mother and son (Group Kingo: N = 1). The two recorded concurrent calling bouts of three individuals consisted of (1) the silverback and his two sons, one a blackback and one a young silverback (Group Buka) and (2) the silverback, his juvenile son, and a female unrelated to the two (Group Kingo).

### Food types and preference categories

During our study period, the two gorilla groups fed on 41 different plant species and two species of insects (Table 2 shows an overview of the recorded gorilla diet for each group). When available, food preference categories are indicated for specific food items. Food-associated calls were produced while feeding on 18 plant and two insect species.

**Table 2:** Foods of the gorilla diet between July and September 2012. Total call frequency and call rates per feeding session are indicated. All fruit was ripe.

Plant species	Local name	Part eaten	Group observed feeding	Known food preference categories indicated: preferred = P, fallback food = F	Total call frequency	Call rate
*Afromomum limbatum*	Injombo	stem	B, K	F	0	0
*Afromomum sp*.	Ndundu *	leaf	B, K		8	0.8
*Afromomum subsericium*	Injokoko	fruit & stem	K		0	0
*Angylocalyx pynaertii*	Monjombe	fruit	K	F	0	0
*Annonidium mannii*	Mobei	fruit	B, K	P	0	0
*Antrocaryon klaineanum/ micraster*	Modiali	fruit	K		0	0
*Aponcynaceae sp*.	Ivua	leaf	K		0	0
*Aquatic herbaceous vegetation*: *Marantaceae*, *Zingiberaceae and Cyperus sp*., including *Hydrocharis chevalieri*, *Nymphaea maculata*, *Rhynchospora corymbosa*	(various swamp vegetation, Konguassika)	leaf, pith, all	B, K	F	4	0.36
*Barteria fistulosa* or *B*. *dewevrei*	Ngumangoma	fruit	K		0	0
*Celtis midlbraedii*	Ngombe	leaf	K	F	0	0
*Chrysophyllum (Gambeya) lacourtiana*	Bambu	fruit	K	F	1	0.25
*Chytranthus macrobotrys*	Motokodi	fruit	K		0	0
*Colletocoema dewevrei*	Mobobo	fruit	B, K		0	0
*Dialium zenkeri* or *D*. *pachyphylum*	Mbaso	leaf	K		0	0
*Diospyros crassiflora*	Lembe	fruit	K		6	0.35
*Diospyros ituriensis*	Babangu *	fruit	B, K		3	0.3
*Diospyros mannii*	Mulombo	fruit	K		0	0
*Duboscia macrocarpa*	Nguluma	fruit	K	F	0	0
*Ficus natalensis*	Ngumu	leaf	K	F	2	0.33
*Ficus natalensis*	Dobo	fruit	K		0	0
*Gilbertiodendron dewevrei*	Bemba *	seeds	B, K	P	71	0.38
*Gnetum africanum*	Koko *	leaf	K	F	5	0.42
*Haumania danckelmaniana*	Basele	stem	B, K	P	3	0.16
*Klainedoxa gabonensis*	Bokoko *	fruit	B, K	P	6	0.43
*Laccosperma secundiflora*	Gao	stem	K		0	0
*Myrianthus arboreus*	Ngata	fruit	B, K		0	0
*Oncoba (Caloncoba) welwitschii*	Isoko	fruit	B		0	0
*Palisota ambigua*	Doto *	stem	B, K	F	5	0.3
*Palisota brachythyrsa*	Mangabo	leaf & stem	B, K	F	0	0
*Pteleopsis hylodendron*	Genye *	fruit	B, K		21	0.23
*Pterocarpus soyauxii*	Mbeli *	flower	B, K	F	5	0.26
*Rubiaceae*, unknown subspecies	Mondaman-daman	leaf	K		0	0
*Sarcophrynium schweinfurthii*	Kaya	fruit & stem	K		0	0
*Sarcophrynium schweinfurthii*	Ngongo	leaf	B, K		0	0
*Sterculiaceae Cola*	Ngaingai	fruit & seeds	K		0	0
*Tetrapleura tetraptera*	Ekombolo *	fruit	B, K	F	11	0.42
*Thomandersia hensii*	Ingooka	leaf	K	F	3	0.3
*Trachyphyrnium braunianum*	Mbonge	stem	K		0	0
*Treculia africana*	Vousa	fruit & seeds	B, K		2	0.33
*Vitex doniana* or *V*. *welwitschii*	Mogweagwea *	fruit	B, K		7	0.23
*Whitfieldia elongata*	Indolu	leaf	B, K	F	3	0.25
**Insect species**						
*Termites*: *Cubitermes sp*.	Kusu	all	B, K		0	0
*Ants*, *unknown species* ^1^		all	B, K		15	0.13

Foods that elicited concurrent calling of two or three individuals are marked with asterisks (next to local name). Where available, food preference categories according to Doran-Sheehy and colleagues (see [32]) are noted as well.

^*1*^ Gorillas frequently engage in soil scratching behavior where they scratch the forest soil with their hands to find edible pieces of food. They are believed to be searching for ants [41] but since the actual ants have never been seen it is possible that the gorillas search for another unknown food item.

**Fig 3:**
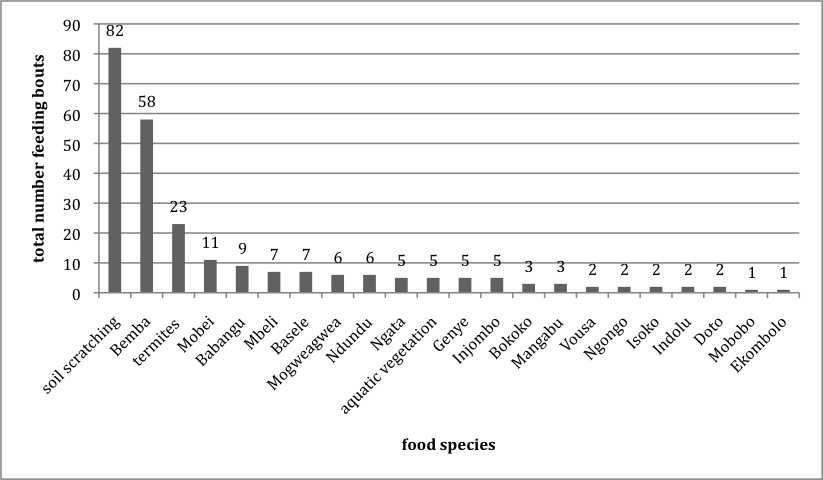
Food bouts recorded from Group Buka, N = 247.

**Fig 4:**
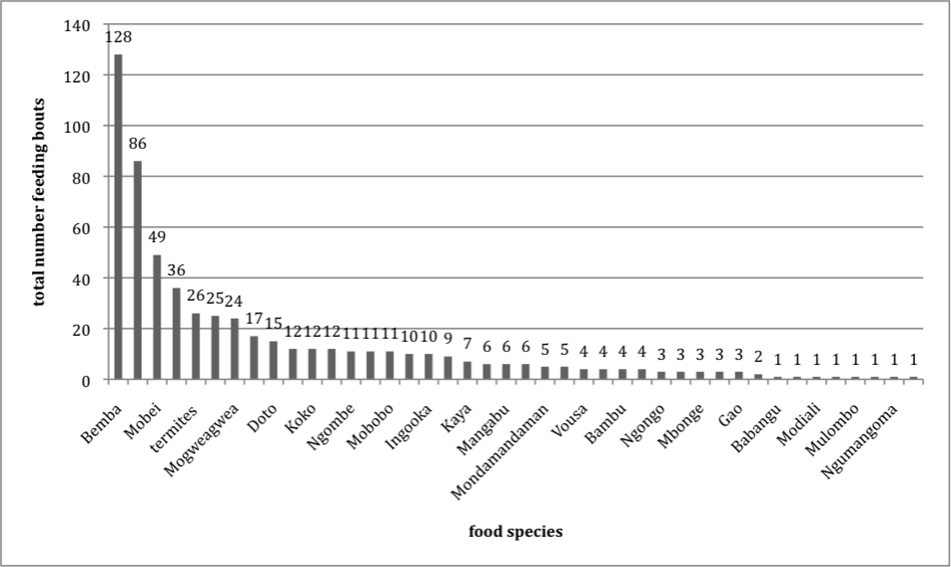
Food bouts recorded from Group Kingo, N = 580.

Our food bout data shows some differences between the two groups concerning food species eaten (see Figs 3 and 4 for an overview of food bouts recorded from both groups). In general, however, the seeds of *Gilbertiodendron dewevrei* (‘Bemba’), the fruit of *Annonidium mannii* (‘Mobei’) (both preferred food items, see [41]), and possibly ants extracted from the forest ground via ‘soil scratching’ accounted for frequent food bouts in each group.

### Model 1: Call probability

Food-associated calls were not equally distributed across all individuals within the two observed gorilla groups. Some individuals never produced food-associated calls during our observation periods: in Group Buka 78% (N = 7) of the investigated group members were recorded engaging in food-associated calling, while in Group Kingo, 55% (N = 6) of investigated group members were recorded producing food-associated calls. Overall, Model 1 showed a clear effect of the factors sex, age, and food type on the probability of producing food-associated calls (full null model comparison). Sex affected calling probability (X^2^ = 11.31, df = 1, P<0.001), with males showing an increased probability of calling as compared to females (estimate+SE: 0.054+0.015). Similarly, age had an effect on calling probability (X^2^ = 13.53, df = 3, P = 0.003), with younger individuals less likely to call (estimate+SE: subadults -0.012+0.021, juveniles -0.040+0.018, infants -0.08+0.02). Food types were shown to affect calling probability (X^2^ = 56.47, df = 6, P<0.001) as well. Aquatic vegetation showed the highest call probability, closely followed by seeds (estimate+SE: -0.007+0.016), whereas insects showed the least probability to elicit calls (estimate+SE: -0.048+0.016).

Preference category of specific foods did not have an effect on calling probability (X^2^ = 0.03, df = 1, P = 0.86). See Table 3 for details of the results.

**Table 3:** Overview of descriptive results.

Age class, sex, food type	Vocalized food bouts/ total nbr. of food bouts	Mean call rate per vocalized food bout	Mean call rate per feeding hour	Food bouts with concurrent calling / total nbr. of vocalized food bouts (%)
Adult	139/ 827	1.92 (±2.1)	0.081 (±0.6)	
Subadult	68/ 827	1.31 (±0.35)	0.06(±0.28)	
Juvenile	18/ 827	1.5 (±0.9)	0.02 (±0.18)	
Infant	0/ 827	0	0	
Female	28/ 827	1.4 (±0.9)	0.01 (±0.14)	
Male	197/ 827	1.76 (±1.84)	0.07 (±0.48)	
Aquatic vegetation	4/ 11	2 (±0.8)	0.11 (±0.49)	2/ 4 (= 50%)
Flower	5/19	3 (±2)	0.077 (±0.55)	0/ 5 (= 0%)
Fruit	74/ 292	1.74 (±1.9)	0.04 (±0.39)	29/ 74 (= 39%)
Insect	17/ 167	1.35 (±0.7)	0.014 (±0.15)	3/ 17 (= 18%)
Leaf	26/ 75	2 (±1.7)	0.06 (±0.44)	10/ 26 (= 38%)
Seed	88/ 187	1.6 (±1.7)	0.07 (±0.5)	36/ 88 (= 40%)
Stem	9/ 76	2.11 (±1.5)	0.03 (±0.3)	2/ 9 (= 22%)
Fallback	52/ 156	1.92 (±1.4)	0.06 (±0.42)	25/ 156 (= 16%)
Preferred	100/ 279	1.71 (±1.75)	0.06 (±0.44)	42/ 279 (= 15%)

### Model 2: Call rate

The call rate was clearly shown to be influenced by the sex (X^2^ = 14.17, df = 1, P<0.001) as well as the age (X^2^ = 16.032, df = 3, P = 0.0011) of the caller. Call rate was higher in males than in females (estimate+SE: 0.06+0.03) and the adult age class displayed the highest call rate in general, with call rate increasing with age (estimate+SE: subadults -0.03+0.04, juveniles -0.07+0.04, infants -0.09+0.02).

The food types had a significant effect on call rate (X^2^ = 35.54, df = 6, P<0.001). Aquatic vegetation elicited the highest call rate, closely followed by flowers (estimate+SE: -0.027+0.04) and seeds (estimate+SE: -0.03+0.03), while insects elicited the lowest call rate (estimate+SE: -0.1+0.03).

Food preference category did not significantly impact the call rate in the observed groups (X^2^ = 0.35, df = 1, P = 0.55). See Table 3 for details.

### Model 3: Probability of more than one caller per food bout

Overall, the probability of multiple callers was clearly influenced by the food type on which the gorillas were feeding (X^2^ = 18.9, df = 6, P = 0.004). Aquatic vegetation displayed the highest probability to elicit concurrent calling bouts, closely followed by seeds (estimate+SE:-0.019+0.01), while insects (estimate+SE:-0.036+0.01) and flowers (estimate+SE:-0.038+0.02) displayed the lowest probability of concurrent call elicitation.

Preference category of the food had no effect on the probability that more than one caller engaged in food-associated calling (X^2^ = 0.013, df = 1, P = 0.91). See Table 3 for details.

## Discussion

The aim of this study is to provide the first detailed analysis of gorilla food-associated calling. We focused our investigation on the behavioral context of food-associated call occurrence as well as aspects related to caller identities. Our results show that food-associated calling occurred exclusively in the feeding context and the majority of food-associated calls were produced during the actual feeding/ingesting activity of particular food items. This is in accord with findings by Salmi and colleagues [27] who recorded humming and singing exclusively in the foraging context. The fact that studies on mountain gorillas describe the vocalizations as also occurring during resting and other contexts [26, 42] may reflect a difference in vocal behavior between mountain and western lowland gorillas (also see [27, 28]). While the vocal repertoire of the two subspecies has been shown to be very similar [27, 28], differences in usage of certain vocalizations may occur due to differences in the ecological conditions or due to innate vocal abilities (see e.g. [27, 43, 44]). An anecdotal report from a captive setting that found food-associated vocalizations to occur in a non-feeding context—during a phase of presumed well-being in a male gorilla [29]—may reflect behavioral differences between captive and free-ranging individuals. Of course, differences in designs of the studies on mountain and western lowland gorillas and captive and free-ranging gorillas may also explain the different results. Additionally, there may be a difference in function and use between the two food-associated vocalizations humming and singing, a fact that remains unexplored by our study. However, Salmi et al. [27] and Hedwig et al. [28], similar to the present study, did not find either of the vocalizations to occur outside the feeding context and it thus seems that the gorilla groups that were investigated for those studies exclusively use the vocalizations during feeding sessions. Similarly, the large majority of food-associated calls of chimpanzees were found to be food-specific [16], further strengthening the claim that food-associated calls are most tightly linked to the feeding context. Generally it has to be mentioned that our results may be an overestimation of the context-specificity of the food-associated calls as we conducted all-occurrence sampling of these calls in feeding contexts and ad-libitum sampling in non-feeding contexts. Ideally, future studies would concentrate on focal follows of subjects and record all of their vocalizations to verify our results.

Our results further show that food-associated call production was not evenly distributed across all individuals within the gorilla groups but was more frequent in adults and males. Call probability as well as call rate were increased in the adult and male age/ sex class. It has to be noted that in western lowland gorillas the vocalizing rate of adult males in general is higher than that of females [27] which may help explain our results. The higher food-associated calling rate could thus be related to a general tendency of males to vocalize more frequently. The question remains as to why this is the case, in particular in the feeding context. Females and immatures are generally at a higher risk of predation and thus behave more quietly [42]. In addition, the lack of social interaction between females may be another relevant factor explaining these vocal differences. The polygynous social structure of gorilla groups may also play a role and cause silverbacks, as leaders of their groups, to vocally coordinate with the other group members on a regular basis [25].

Concerning food types, we measured an effect on the call probability as well as the call rate. Food types had an effect on the probability of calls being given as well as the call rate. Aquatic vegetation, flowers, and seeds led to a high likelihood of call production. Insects, in particular, showed the lowest probability and rate of calling. In gorillas, similar to bonobos and chimpanzees, food-associated calling behavior is dependent on the food source to some extent [18, 45]. An important next step would be to acoustically evaluate each humming and singing instance to determine whether subtle acoustical cues distinguish food-associated calling in particular feeding contexts, as has been done by Slocombe and Zuberbühler for chimpanzees [18] and Clay and colleagues for bonobos [45]. Furthermore, the probability of multiple individuals engaging in concurrent calling during feeding bouts was also influenced by the food types on which the gorillas were feeding. Contrarily, food preference categories did not have an impact on calling behavior in the feeding context, however, it has to be kept in mind that food preference categories were not available for all of our feeding data. In addition, preferences may be more varied on the individual as well as group level, which could be revealed by more fine-grained preference analyses.

Although not directly tested in this study, we describe how our findings relate to existing hypotheses of food-associated call function. We outline the steps future studies could take to test these ideas. One possible function of call production during feeding could be intra-group coordination (the “coordination hypothesis”, see [8]. As the food-associated calls of gorillas are relatively quiet, it does not seem feasible that gorillas would use them for communication over longer distances. This also makes the food-associated calls unlikely candidates for food-advertisement signals, where other group members are informed of a food discovery (or “food information hypothesis”, see [6, 7]. Furthermore, food-associated calling occurred mainly *in the course of* food consumption, i.e. while the whole group was already feeding on a particular food source, rather than at the beginning of feeding or upon initial discovery of food. In addition, in a typical food-associated call event all group members were in relative proximity to one another, making food advertisement redundant. Food-associated calling may function to notify the rest of the group of an individual’s current feeding activity, similar to what has been reported for chimpanzees [8]. Informing other group members of one’s current activity could be important for group coordination and cohesion. Such a scenario could also explain the higher frequency of adult male calls: Silverbacks may have to engage more frequently in auditory informing as they are generally the ones initiating changes in group activity [25]. Close-range vocal production during feeding may provide the nearby group members with the information that feeding is ongoing; cessation of food-associated calling may indicate that a change in group activity is imminent. Under such a premise, the lower frequency of immature calling may also be explained: the fact that younger individuals do not play a role in group decision-making may preclude them from the necessity of informing others about their current activity. Similar factors may apply to female food-associated calling. An intra-group function of gorilla food-associated calling might be the spacing and coordination of feeding within a food patch, as in capuchin monkeys [7, 46]. In order to test the coordination hypothesis on the food-associated calls of gorillas, the crucial questions concern whether gorillas call more when feeding on dispersed food patches with individuals out of sight of one another than when they are clumped together in closer proximity and in visible contact. Assessing the exact proximity of individuals during food-calling sessions would be an important next step to determine if the calls are related to intra-individual feeding behavior and spacing in order to draw conclusions as to a coordinative function of the calls.

Alternatively, sense of well-being in general may elicit food-associated call vocalizations in gorillas, rather than solely the act of feeding [29]. The fact that specific foods elicited more calling and simultaneous calling of multiple individuals may indicate that attitude toward (possibly contentment over) the food source plays some role in the production of food-associated calling in gorillas. Food-associated calling could be an expression of well-being in gorillas when feeding on specific, preferred foods. Even though the influence of preference category of food types did not reach significance with regards to food call probability, a larger sample size combined with more fine-grained, individual preferences of the individuals may yield different results. This “well-being hypothesis”, however, is not mutually exclusive with the “coordination hypothesis” [8]. On a proximate level, food-associated calling might reflect emotional well-being triggered by the consumption of particular food. At the same time, food-associated calling might also function to signal the act of feeding itself to listeners.

Gorilla food-associated calling is an interesting phenomenon for various reasons. Firstly, the vocalizations’ function does not seem to be congruent with what has been described for the majority of primate food-associated vocalizations. While the advertisement of a food discovery to other, ignorant group members plays an important role in many primate species, the food-associated calls of gorillas seem to be more tightly related to dynamics of intra-group coordination during feeding sessions. Food-associated calling could represent a form of collective decision-making in the feeding context and allow group members to coordinate their feeding activities [5, 7]. It seems that, while many other primates use their food-associated calls to alert others, gorillas and chimpanzees [8] may use theirs to inform the other group members of the fact that they are still in the process of feeding.

Food-associated calls of gorillas represent an interesting avenue for the investigation of great ape vocalizations concerning the individual as well as the group level. Deeper insights into the degree of individual and group-specific variation in form and function, acquisition and learning mechanisms, and the exact structuring of humming and singing could not be explored by the present study, however, we hope to inspire future studies that aim to investigate these variables in the food-associated calling of gorillas.

## Supporting Information

S1 File Balema singing (Gilbertiodendron dewevrei). (WAV)

S2 File Mobimba singing (Gilbertiodendron dewevrei). (WAV)

S3 File Kusu humming (Pteleopsis hylodendron). (WAV)

S4 File Kusu humming (Pteleopsis hylodendron). (WAV)
